# THE STANDARD POSTURE IS A MYTH: A SCOPING REVIEW

**DOI:** 10.2340/jrm.v56.41899

**Published:** 2024-10-15

**Authors:** Martin E. BARRA-LÓPEZ

**Affiliations:** 1 International University of Catalonia, Sant Cugat del Vallès, Barcelona, Spain

**Keywords:** gravity line, head forward posture, plum line test, posture assessment, standard posture

## Abstract

**Background:**

The standard posture described in Kendall’s manual is commonly used for postural assessment. However, no bibliographic reference was provided to support its use.

**Objective:**

To identify the original source and the procedure followed for the design of that posture and to compare it with current literature on the subject.

**Methods:**

In accordance with the PRISMA Extension for Scoping Reviews recommendations, PubMed and Scopus were searched using the terms “standing posture”, “plum line,” and “gravity line”. Publications in English, French, German, or Spanish that referred to posture in adults without pathology were included.

**Results:**

Six articles and 3 books were included in the final analysis. An identical posture to that described in Kendall’s manual was identified in an early 19th-century work carried out with the unrealistic objective of maintaining static bipedal standing without muscular support, and including several anatomical misconceptions. Furthermore, the “ideal alignment” described in Kendall’s manual does not correspond to the actual line of gravity, the comfortable posture, or natural postural compensations due to age, gender, or race.

**Conclusion:**

The utilization of this standard to ascertain postural deficiencies is not supported by current evidence and may result in numerous false positives, particularly in the elderly.

## LAY ABSTRACT

The assessment of postural deviations is a common practice in physiotherapy and rehabilitation clinical settings. A standard was described in the literature, and the plumb line test was used to compare it with the current posture of the subject. Still, this scoping review has identified that this standard was developed in the earlier 19^th^ century under an unrealistic objective and included several anatomical misconceptions. Even more, in asymptomatic subjects, that standard does not correspond to the actual line of gravity, a comfortable posture, or natural postural compensations due to age, gender, or race. Using this standard to ascertain postural deficiencies may result in numerous false positives, particularly in older adults. In clinical practice, posture assessment must be based on a global, individualized examination, including the subject’s natural posture, rather than being subordinated to a particular standard of comparison.

Postural assessment is widely used in both physical therapy and rehabilitation clinical settings, because of the widespread assumption that poor posture, such as head forward, is associated with pain. There are a number of techniques that can be used to assess a subject’s posture. While these techniques are generally considered reliable, their validity or sensitivity remains uncertain in many instances (1).

The plumb line test, first described by Kendall et al. (2), is a common clinical test used to assess posture while standing (3) and sitting (4, 5). In addition, Kendall’s manual describes the “standard posture” in which the body segments are in “ideal alignment” in a sagittal plane when the plumb line passes through the external auditory canal, midway through the shoulder, slightly posterior to centre of hip joint, slightly anterior to axis of knee joint, and slightly anterior to the lateral malleolus of the ankle (6).

Nevertheless, it is noteworthy that no edition of Kendall’s manual provides a bibliographic reference or details of the procedure used to establish these reference points as the “ideal alignment” or the resulting posture as the “standard posture”.

As evidence-based clinical practice advocates for using the best evidence (in conjunction with clinical expertise and patient values) to guide healthcare decisions, this scoping review aims to identify the original source and the procedure followed for the design of that “standard posture” and to compare it with the current literature.

## METHODS

### Study design

This scoping review was conducted in accordance with the recommendations of the PRISMA Extension for Scoping Reviews (PRISMA-ScR): Checklist and Explanation (7). A 5-step strategy was followed: (*i*) identification of the research question, (*ii*) identification of relevant studies, (*iii*) selection of studies, (*iv*) extraction of information, and (*v*) reporting of results (8).

### Data sources and searches

The search strategy was developed in 3 steps (9). In the first step, a search was carried out in PubMed and Scopus using the term “standing posture” in English, French, German or Spanish, without limiting the publication date or article type and applying the filter “humans’”. After analysing the terms found in the titles and abstracts of the relevant articles, “plumb line” and “gravity line” were also identified as relevant search terms. A second search was carried out in the same databases using these terms and the same filters. The third step was to examine the references in the selected articles and in the first edition of the book *Posture and Pain*.

### Eligibility criteria

The inclusion criteria were articles or books without a publication date limit that discussed posture in adults (>18 years) without pathology. The exclusion criteria were articles that did not include the entire body, referred to specific professions/tasks, or referred to different postural assessment instruments/methods.

### Studies selection

The titles and abstracts of the retrieved studies were assessed, and those that did not meet the inclusion and exclusion criteria were discarded. The remaining potential studies were retrieved in full text and analysed in detail to decide on their inclusion. In addition, all references cited in the selected studies and in the first edition of the book *Posture and Pain* were also examined to identify additional literature.

### Data analysis and synthesis

The included literature was subjected to a comprehensive analysis by the author. The concepts and objectives followed by the different authors for the definition of the analysed postures, as well as the measurement of the differences between the standard posture and the actual line of gravity or the natural posture of the subjects, were synthesized in order to identify critical concepts concerning the posture recommended in Kendall’s manual as a reference for the determination of postural abnormalities.

## RESULTS

### Selection of sources of evidence

**Fig. 1:**
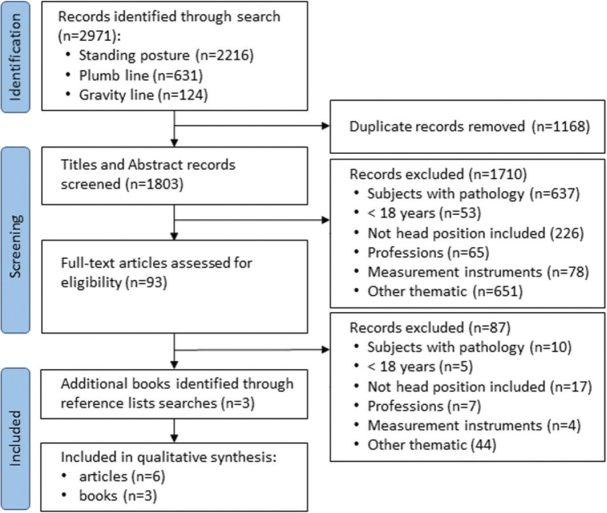
PRISMA flow diagram of the search and selection process.

The search process and the selection of studies, with reasons for exclusion, are presented in a PRISMA flow diagram (Fig. 1). The search strategy yielded a total of 2,971 results. Following the removal of duplicates and the screening of titles and abstracts, 93 full-text articles were reviewed. Based on a detailed reading of these articles, 87 were excluded, leaving 6 articles selected. One book (10) was selected from 1 of the articles, and another (11) was chosen from the references cited in the first edition of *Posture and Pain*. The reading of this second book enabled the identification and selection of a third book of interest (12). Consequently, the bibliography for this scoping review comprises 6 articles and 3 books.

Table I provides an overview of the included manuscripts, indicating the type of document, the sample size and mean age where appropriate, and the main conclusion regarding the alignment described in Kendall’s manual.

**Table I:** Characteristics of included studies

Author (year)	Study / Document	Subjects (Men–Women)	Mean age (rang)	Main conclusion with respect to the standard position described in Kendall’s manual
Brunnstrom S. (1954), 20	Bibliographic revision	-	-	The “perpendicular” posture standard may have arisen from a misinterpretation of the work of Braune and Fischer … and who, unfortunately, chose to call this posture “normal stance”
Griegel-Morris P, et al. (1992), 11	Cohorts	88 (41–47)	32.5 (20–50)	A high incidence of postural abnormalities (forward head=66%, kyphosis=38%, right rounded shoulder=73%, left rounded shoulder=66%) was observed in the Thoraco-Cervical-Shoulder region in a group of healthy subjects between the ages of 20 and 50 years
Gangnet N, et al. (2003), 33	Descriptive	34 (16–18)	29.1 (19–48)	The line connecting the middle of the external acoustic meatus to the middle of the bi-coxofemoral axis was almost vertical. Its mean distance to the gravity line was 30 mm. Our data show a left lateralization, with respect to the gravity line, of the Head-Spine-Pelvis segments. The mean distance was 7.6 mm
Hasegawa K, et al. (2017), 29	Descriptive	136 (40–96)	39.7 (20–69)	Our results revealed that ageing induces trunk stooping, but the global alignment is compensated for by an increase in the cervical lordosis (CL), pelvis tilt (PT), and knee flexion, with the main function of CL and PT to maintain a horizontal gaze in a healthy population
Xue et al. (2020), 34	Cohorts	50 (25–25)	Unknown (21–30)	Significant differences in sagittal radiographic parameters were found between the standard upright position and the natural and comfortable upright position; the latter served as a marker for energy conservation during standing and revealed a more kyphotic spinal profile
Ouchida J, et al. (2023), 35	Cohorts	317 (125–192)	43.8 (20–84)	While several local alignment changes with age were found, changes in global parameters related to the centre of gravity were kept relatively mild by the chain of compensation mechanisms including the lower limbs
Braune W, Fischer O. (1889), 17	Book	Title: Über den Schwerpunkt des menschlichen Körpers mit Rücksicht auf die Ausrüstung des deutschen Infanteristen		
Steindler A. (1935), 18	Book	Title: Mechanics of Normal and Pathological Locomotion in Man		
Weber W, Weber E. (1836), 19	Book	Title: Mechanik der menschlichen Gehwerkzeuge. Anatomisch–physiologische Untersuchung		

### Historical background

The first scientific attempt to ascertain the position of the centre of gravity of the human body was carried out by Giovanni Alfonso Borelli (in the 17^th^ century) (13). His methodology was capable only of calculating its height in the frontal plane, not its depth in the sagittal plane. Additionally, Borelli was the first to propose the notion that to maintain balance: “The line perpendicular to the plane of the horizon passing through the centre of gravity must fall between the soles or on the sole of one foot so that the bony pillars of our limbs can support the whole weight without the help of the muscles” (13).

In the 19^th^ century, there was a growing interest in upright posture due to the supposed association between stooped posture and various systemic diseases (14). The brothers Wilhelm and Eduard Weber reproduced Borelli’s method of locating the body’s centre of gravity in the frontal plane. They also supported his concept that the bones of the lower limbs could support the weight of the whole body without the help of the muscles if all the segments of the body were in balance. Accordingly, they designed a posture for this purpose (12).

Other authors have also designed bipedal postures following different methodologies (15), but the most notable work in this area was carried out by Braune and Fischer (10, 16). Their aim was to improve the ergonomics of German infantry equipment, and to this end: “Primarily we had one goal: to find a natural initial attitude that would be appropriate for measurements and calculations” (16).

They were the first to locate the centre of gravity using a 3D method, suspending the whole human body and each of its parts separately in the 3 planes of space. To facilitate subsequent calculations, they designed a posture in which all the centres of gravity (except for the foot, for obvious reasons) were located on the same vertical line passing through the joint axes of the hips and knees. A soldier adopted this stance without losing his balance, so the authors considered it a natural stance, which they called “normalstellung” (normal stance) (10). From this “norm”, they were able to carry out precise geometric constructions and efficient mathematical calculations in other positions, such as the firing position.

The supposed link between posture and health was emphasized by Joel E. Goldthwait in his historic speech at the Massachusetts Medical Society (Boston) in 1915, entitled An Anatomical and Mechanistic Conception of Disease (17). Goldthwait stated: “When used rightly, or fully erect, the feet, knees, hips, spine, shoulders, head and all the portions which represent the body’s frame, are used in balance, with the greatest range of movement possible without strain” (17).

Until the middle of the 20th century, poor posture, i.e., any posture that was not upright, was associated with various cardiac, respiratory (e.g., tuberculosis), digestive, or psychiatric diseases (18, 19). However, as knowledge regarding these diseases increased, posture was no longer considered a causative factor. From the 1950s onwards, there has been increasing interest in the relationship between posture and pain (20).

In 1952, Dr Henry O. Kendall and the physiotherapists Florence P. Kendall and Dorothy A. Boynton published a book with the unequivocal title *Posture and Pain* (2). The Kendalls also authored the acclaimed manual *Muscles, Testing and Function* (21). In later editions, they integrated both tests into a single book that remains a reference manual for clinical practitioners and has recently been reissued and updated (6).

### The “standard posture”

Several authors have attributed the authorship of the posture used as a reference for assessing postural deviations to Braune and Fischer (5, 22). Signe Brunnstrom also criticized the term “normalstellung”, noting that it could be interpreted as suggesting that a perpendicular posture is intrinsically desirable. She wrote: “Obviously, perpendicular posture does not coincide with Nature’s way of balancing the body and, therefore, should not be used as a standard for good posture” (23). In any case, the posture developed by Braune and Fischer is not exactly the same as that described in Kendall’s manual.

Moreover, in the first edition of *Posture and Pain* (2), citing Steindler’s criteria (11), postures such as those of Braune and Fischer were explicitly rejected because of the instability implied by the line of gravity passing precisely through the joint centre of the hips and knees. Steindler also claimed that the definition of a standard or pathological posture must encompass not only its morphology but also the gravitational stress it is subjected to, and he also lauded the work published by the Weber brothers in 1836 (12).

It is reasonable to assume that the Kendalls adopted the posture described by the Weber brothers, which in theory has no gravitational stress, as only Steindler was cited on this point in the first edition of *Posture and Pain*. Regardless, the so-called “standard posture” is identical to the Weber brothers’ design.

As previously stated, the Weber brothers wanted to devise a posture that would maintain a balanced bipedal stance, following Borelli’s erroneous hypothesis that muscular assistance is not necessary to support the weight of the entire body if all the body segments are balanced over the bones of the lower extremities. To achieve this, they postulated that the centre of gravity was located in a frontal plane between the femoral heads. The line of gravity was assumed to be slightly behind the centre of rotation of the hip joint and in front of the centre of rotation of the knee joint. In this configuration, the bodyweight carries the hip and knee joints in extension, limited in both cases by ligamentous tension.

With regard to the head, unlike the lower limbs, no ligamentous support was expected, because according to anatomical ideas (24–26,) of the era: “The nuchal ligament is almost totally absent in man and can be dispensed with because his head, when carried erect, is supported vertically under his centre of gravity and his weight, therefore, only presses on the bony base on which it rests” (12). Accordingly, they stipulated that the head should be held in an erect position with its centre of gravity, which projects into the external auditory canal (27), aligned vertically with the odontoid process (C2), and in the same line of gravity devised for the lower extremities. The upper limbs were placed in the same line of gravity by moving the shoulders back.

**Fig. 2:**
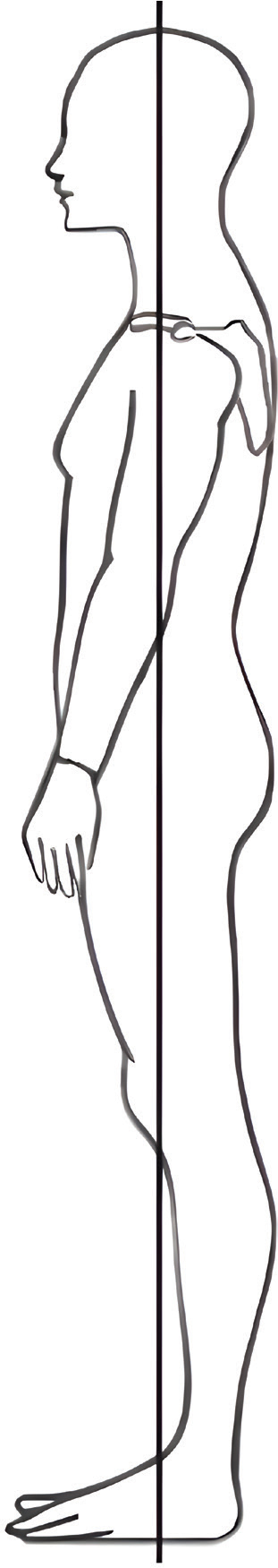
Drawing of ideal alignment, as described in Kendall’s manual.

At no point did the Weber brothers mention that the position they devised should be considered a reference position (28). However, Kendall’s manual refers to it as the “standard posture” and the “ideal posture” (Fig. 2). Nevertheless, it was designed on the unrealistic assumption of static bipedal balance, whereas standing is known to be inherently unstable (29); it is based on an anatomical misconception of the nuchal ligament, and assumes a recurvatum of the knees which, however slight, cannot be considered standard (30).

Of this “ideal”, in the first edition of *Posture and Pain*, the authors assert: “It involves the position and alignment of so many joints and parts of the body that it is not probable that any individual can meet this standard in every respect. As a matter of fact, the authors have not seen an individual who matches the standard in all respects” (2). Surprisingly, this important statement disappears in subsequent editions.

### Actual gravity line

The gravity line is defined as the vertical to the centre of pressure recorded using a pressure platform (31). The centre of pressure represents the projection of the subject’s centre of gravity onto the ground (32). The displacement of the centre of pressure is considered a measure of body sway (33). The centre of pressure can also be recorded during a radiological assessment, and the gravity line can be projected onto the radiological images to delineate the corresponding anatomical landmarks (34).

Gangnet et al. (35), using a force platform and a 3D radiographic study, found that the centre of the femoral heads was 28 mm anterior to the line of gravity. The line joining the centre of the acoustic meatus and the centre of the femoral heads, which is not strictly vertical, is anterior to the line of gravity, and the mean distance between the two is 30 mm. In the frontal plane, the head–pelvis line is displaced 7.6 mm to the left of the line of gravity due to the difference in body mass on either side of the sagittal plane.

Hasegawa et al. (31) found that the hip axis is on average 14 mm anterior, the knee axis 24 mm posterior, and the ankle 48 mm posterior to the line of gravity. Overall, their position is not a vertical line. Their study shows that ageing causes the trunk to stoop, and that the global alignment is compensated for by an increase in cervical lordosis, pelvic tilt, and knee flexion, allowing the healthy population to maintain a horizontal gaze.

Xue et al. (36) performed whole-body radiographs to determine the differences between standard and comfortable natural postures. All spinal curvatures and the relationship of the spinal axis to the hip, knee, and ankle joint centres show statistically significant differences between the 2 postures, with the natural posture being more kyphotic overall. The authors conclude that the natural and comfortable upright position served as a marker for energy conservation during standing.

Ouchida et al. (37) analysed various parameters of whole body and segmental sagittal alignment by radiological examination in the largest sample of the included studies. His data show changes in the alignment of different body segments with age. However, for the centre of gravity, the changes in global parameters are smoother thanks to the chain of compensatory mechanisms involving the spine (e.g., increased cervical lordosis) and the lower limbs (increased knee flexion), with the ultimate aim of maintaining the horizontality of gaze.

Griegel-Morris et al. (3) assessed the postural abnormalities in 88 asymptomatic subjects without pathology using the anatomical references of the standard posture described in Kendall’s manual. Although they considered it normal if the head or shoulders were less than 1 cm in front of the plumb line, they found 66% of subjects with a forward head, 38% with kyphosis, 73% with a forward right shoulder, and 66% with a forward left shoulder.

## DISCUSSION

The “standard posture” described in Kendall’s manual is identical to that developed by the Weber brothers in 1836. They mistakenly aimed to develop a posture based on Borelli’s myth that static standing is possible without muscular work.

It is also important to note that the Weber brothers designed this posture using an unreal line of gravity and based on an anatomical misconception concerning the nuchal ligament. Contrary to the beliefs of 19^t^h-century anatomists, the nuchal ligament has a complex structure (38, 39), limits movement in the transverse and sagittal planes (40, 41), and maintains cervical lordosis (42).

Kendall’s manual states that the erect head is a position of equilibrium that minimizes the stress on the cervical musculature (6). However, electromyographic studies have shown that, from a certain degree of cervical flexion, the dorsal musculature becomes more relaxed, to the point of electromyographic silence (43, 44, 45).

Kendall’s manual emphasizes that the “standard posture” is an “ideal posture”, but in the first edition of *Posture and Pain* the authors admitted that they had not seen any individual who met all their requirements. Consequently, compared with this “ideal”, most subjects show postural irregularities, especially head or shoulder forward posture (46), even in asymptomatic subjects (3). The question arises as to the extent to which pathological alignments are over-diagnosed, when in fact they are simply differences from a model designed for an unrealistic purpose based on anatomical misconceptions.

**Fig. 3:**
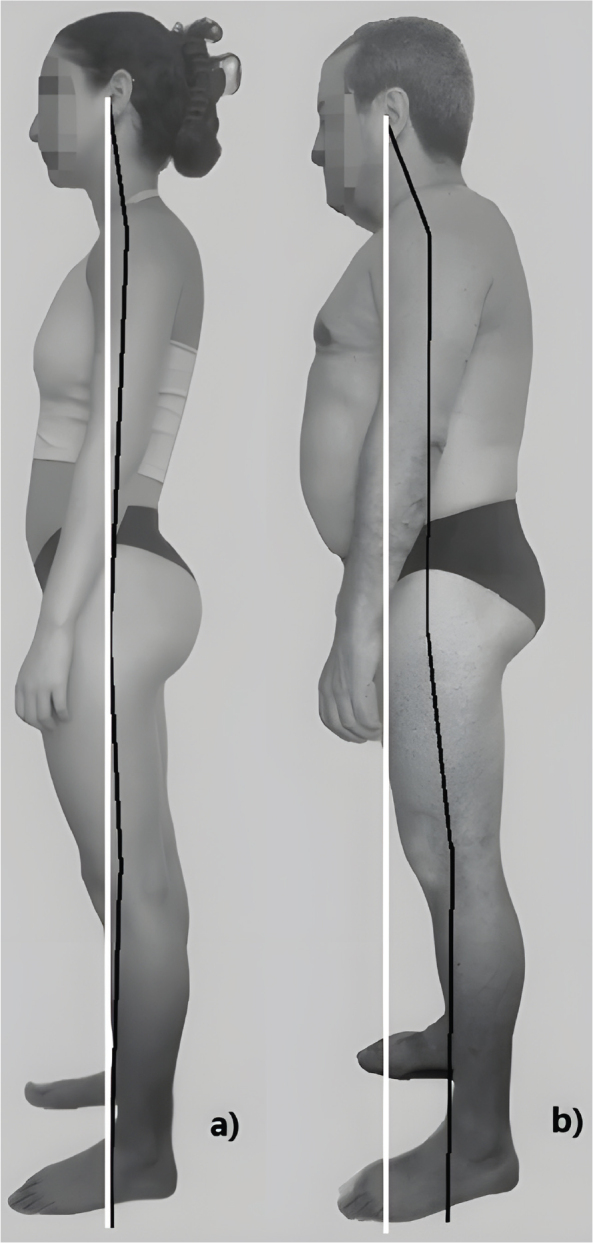
Discrepancies between natural upright posture and the “ideal alignment” in (a) a 22-year-old woman and (b) a 68-year-old man, both of whom are asymptomatic and have no history of musculoskeletal conditions.

In addition to large intersubject variability (34, 47–49), the natural posture changes with age (31, 37, 50–52), moving further and further away from the “ideal alignment”, although in different ways according to race (53), so that the likelihood of false-positive deformities increases with age (Fig. 3). The postural compensations that older, healthy subjects naturally adopt are essential for maintaining gaze in the horizontal plane (31). Although the cervical spine is the most involved segment (31, 37, 54), the chain of compensation also involves the lower limbs. If one of these segments is experiencing pain, attempting to correct its position would only make sense if the entire chain of compensation could be involved so as not to alter the horizontality of the gaze.

Neither the natural posture nor the actual line of gravity corresponds to the “ideal alignment” described in Kendall’s manual. In the natural posture, the spine is more kyphotic (36); the line of gravity does not pass through the centre of the frontal plane (35, 55, 56), the acoustic meatus does not form a vertical axis with the femoral head (31, 35, 56), the centre of pressure does not lie close to the lateral malleolus (31), and the anatomical landmarks of the “standard posture” are not aligned (31, 57, 58).

In none of the included radiological studies was it possible to assess the position of the shoulders in relation to the standard. Although changing the position of the arms causes changes in the line of gravity (56), subjects are asked to place their upper limbs in front of the chest to allow a better view of the spinal statics (36).

There is widespread belief that poor posture causes pain. The literature shows a correlation between postural irregularities and pain (3, 59, 60) or reduced quality of life (61). However, a statistical correlation does not prove causation, and there is also literature that contradicts this association (60, 62, 63).

In all the editions of *Posture and Pain*, the authors discuss that subjects with severe postural abnormalities may not experience pain, while those with good posture may experience severe pain. From the first edition of their manual, the authors attribute these discrepancies “to the constancy of the defect” (6) and suggest that, in the first case, the subject is likely to change posture frequently and, in the second case, the subject may have a stiffness that does not allow him to change his posture. This leads to the question of whether the pain is a consequence of the posture itself or of maintaining it over time.

Korakakis et al. (64) show the lack of complete consensus among physiotherapists concerning good posture both standing and sitting; however, they fund a widespread tendency to consider a non-forward head posture or a neutral lumbar lordosis as positive characteristics despite the lack of robust evidence that any particular posture is associated with better health outcomes. As the attitudes and beliefs of health professionals, together with those of patients, can influence healthcare (65), clinical practitioners need to be aware of the assumption that some postural variabilities, such as forward head or shoulders, do not correspond to good posture is based on 19^th^-century postural and anatomical misconceptions.

Postural evaluation must be based on an individual assessment that cannot be subject only to a single standard of comparison. In addition, the natural posture of the subject must be taken into consideration (36). Moreover, it does not seem reasonable that the solution to our patient’s complaints was to adopt a supposedly ideal posture designed on the basis of an unrealistic objective, including anatomical misconceptions, and whose proponents acknowledged that they had never observed an individual who met this standard in all respects.

### Limitations

This scoping review was limited to only a few languages and excluded articles published in other languages. A single author was responsible for the screening, inclusion, exclusion, and data extraction. The methodological quality of the included studies was not critically appraised according to the guidelines for conducting scoping reviews.

### Conclusion

The alignment considered as a postural standard is questionable for several reasons. Primarily, it was designed based on a mythical and unrealistic objective, and it includes some anatomical misconceptions. Second, it is a single model that does not account for natural variations due to age, gender, or race. Third, current evidence does not support its use. The utilization of this standard to ascertain postural deficiencies may result in numerous false positives, particularly in the elderly. Posture assessment must be based on a global, individualized examination that cannot be subordinated to a particular standard of comparison.
